# Machine Learning-Based Heavy Metal Ion Detection Using Surface-Enhanced Raman Spectroscopy

**DOI:** 10.3390/s22020596

**Published:** 2022-01-13

**Authors:** Seongyong Park, Jaeseok Lee, Shujaat Khan, Abdul Wahab, Minseok Kim

**Affiliations:** 1 Department of Bio and Brain Engineering, Korea Advanced Institute of Science and Technology (KAIST), Daejeon 34141, Korea; sypark0215@kaist.ac.kr (S.P.); shujaat@kaist.ac.kr (S.K.); 2 Department of Mechanical System Engineering, Kumoh National Institute of Technology, Gumi 39177, Korea; leelion7027@kumoh.ac.kr; 3 Department of Aeronautics, Mechanical and Electronic Convergence Engineering, Kumoh National Institute of Technology, Gumi 39177, Korea; 4 Department of Mathematics, Nazarbayev University, Nur-Sultan 010000, Kazakhstan; abdul.wahab@nu.edu.kz

**Keywords:** surface-enhanced raman spectroscopy (SERS), machine learning, heavy-metal ion, neural network, SVM, random forest, pattern classification

## Abstract

Surface-Enhanced Raman Spectroscopy (SERS) is often used for heavy metal ion detection. However, large variations in signal strength, spectral profile, and nonlinearity of measurements often cause problems that produce varying results. It raises concerns about the reproducibility of the results. Consequently, the manual classification of the SERS spectrum requires carefully controlled experimentation that further hinders the large-scale adaptation. Recent advances in machine learning offer decent opportunities to address these issues. However, well-documented procedures for model development and evaluation, as well as benchmark datasets, are missing. Towards this end, we provide the SERS spectral benchmark dataset of lead(II) nitride (Pb(NO_3_)_2_) for a heavy metal ion detection task and evaluate the classification performance of several machine learning models. We also perform a comparative study to find the best combination between the preprocessing methods and the machine learning models. The proposed model can successfully identify the Pb(NO_3_)_2_ molecule from SERS measurements of independent test experiments. In particular, the proposed model shows an 84.6% balanced accuracy for the cross-batch testing task.

## 1. Introduction

The contaminants in water are usually complex mixtures of compounds whose detection requires analytical chemistry techniques such as sampling, purification, separation, and quantification using special instruments such as the High-Performance Liquid Chromatography (HPLC) and Gas Chromatography (GC). In general, these analytical chemistry methods exhibit high sensitivity, specificity, and precision, but require expertise because complex instrumentation and analytical procedures are needed. Therefore, it is not very feasible to apply these technologies that require low-cost, light-weighted portable applications operated by non-professionals. To this end, a method for detecting contaminants using the SERS has been actively proposed recently (see, for instance, Bodelon et al. [1]).

Surface-Enhanced Raman Spectroscopy (SERS) is a commonly used sensing technique that shares the advantages of conventional Raman spectroscopy, such as easy sample preparation, molecular fingerprinting, and low signal attenuation by solvents while improving sensitivity. Specifically, the surface of the SERS device is usually coated with metal nanoparticles, which induces surface plasmon resonance localized on the metal surface to amplify the Raman scattering signal of the target molecule by up to $10^{8}$ or more (see, for instance, Langer et al. [2]). Hence, SERS provides greater system design flexibility than Raman spectroscopy, making it suitable for portable applications such as heavy metal detection in water [3].

Raman spectroscopy is a widely used sensing technique. Numerous applications of Raman spectroscopy have been reported in the fields of bioanalysis [4], medicine [5], and materials science [6]. For example, Kuang et al. [7] reported the use of Raman spectroscopy to investigate the protein dynamics of membrane proteins. Ho et al. [8] reported rapid identification of pathogenic bacteria using Raman spectroscopy. Calizo et al. [9] utilized Raman spectroscopy to characterize the temperature dependence of graphene. Despite success in reported studies, it is well-known that the weak Raman signals of some target analytes such as bacterial cells and the strong fluctuations in Raman spectroscopy and SERS make it difficult to design reliable and repeatable sensing techniques [10].

The Raman spectra of the SERS measurements show strong variations in intensity and spectral profiles due to the interaction between the molecule and the surface of the SERS device. Therefore, advanced fabrication techniques are required to enhance the identifiability of the target molecule from the spectral dataset. For example, heavy metal ion detection using the SERS itself is a challenge since heavy metals generally have small Raman cross-sectional areas and low adsorption to metal surfaces [11]. Towards this end, many methods have been proposed for improving the adsorption of heavy metal ions using carbon nanotubes or for amplifying the Raman scattering signal by creating hot spots integrated with metal nanoshells [12]. Unfortunately, these complex nanopatterning-based Raman scattering signal amplification methods inevitably lower the reproducibility of the SERS measurements and make it difficult to establish standardized protocols. Moreover, they only guarantee satisfactory performance for specific target molecules, thus, stymieing the applicability of the device [13].

Machine learning has been effectively used recently for numerous applications, including SERS-based molecular detection [14,15,16,17,18,19,20]. In fact, applying machine learning techniques on the spectroscopic dataset is not new. For example, Malitckii et al. [21] applied an Artificial Neural Network (ANN) on hydrogen Thermal Desorption Spectroscopy (TDS) data to characterize steels susceptibility to hydrogen embrittlement. Sbirrazzuoli et al. [22] proposed an ANN model that can identify the structure of an unknown molecule by infrared spectroscopy. Güven et al. [23] proposed an Support Vector Machine (SVM) model that can combine functional near-infrared spectroscopy and Electroencephalography (EEG) measurements signal for the diagnosis of attention-deficit hyperactivity disorder. Guillén et al. [24] proposed an Radial Basis Function kernel (RBF) neural network using near-infrared spectroscopy to classify white and Iberian pork. Janani et al. [25] proposed an Independent Component Analysis (ICA)-based Brain-Computer Interface (BCI) application of functional near-infrared spectroscopy signals. Park et al. [26] proposed a neural network model for detecting R6G molecules. These examples illustrate the successful implementation of machine learning techniques on the spectroscopy dataset.

Motivated by these successes, we design a machine learning application for heavy metal ion detection in the SERS dataset. Since the task of heavy metal ion detection is concerned with the determination of the number of target heavy metal ions subject to a threshold defined by a regulatory body, the underlying problem can be formulated as a binary classification problem. Accordingly, supervised binary classification models have been frequently used for tailored problems using the SERS measurements. Unfortunately, the performance of supervised binary classification models on the SERS datasets is strongly affected by the preprocessing and evaluation methods; see, for instance, the review article on machine learning applications using the SERS measurements [27]. Despite successful applications of machine learning and deep neural network-based learning in the SERS spectrum, an in-depth understanding of the relationship between data collection, preprocessing, and model evaluation is lacking. This understanding is critical for precisely explaining the results as most of them are obtained from vague definitions of the independent test datasets. Towards this end, it is crucial to develop benchmark datasets and performance evaluation criteria to guide the development of machine learning-based heavy metal ion detection in the SERS datasets.

In this article, we propose a machine learning-based heavy metal ion detection model using the SERS measurements. To train the model, we first design a new benchmark dataset for heavy metal ion detection tasks in the SERS measurements. We use lead(II) nitride (Pb(NO_3_)_2_) as our target molecule since it is a well-characterized and widely-used molecule in the SERS-based heavy metal ion detection applications [3]. Then, we conduct an extensive Explanatory Data Analysis (EDA) on the SERS dataset to provide an insight into the relationship between different preprocessing techniques and the performance of various machine learning methods for the SERS-based molecule detection tasks. The performance of the trained model is evaluated on similarly constructed but independently measured SERS spectra.

The contents of the article are arranged as follows. The material and method used in this study are discussed in Section 2, followed by a detailed analysis of the experimental results in Section 3. Finally, the conclusions are drawn in Section 4.

## 2. Materials and Methods

In this section, we provide a detailed description of the proposed SERS-based heavy metal ion detection framework.

### 2.1. SERS Measurements

**Figure 1:**
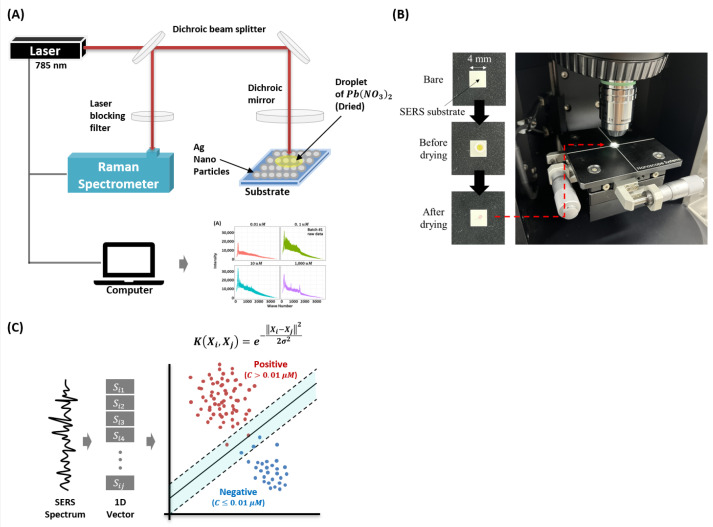
Configuration of the study. (**A**) Experimental setup of the proposed surface-enhanced Raman spectroscopy-based Pb(NO_3_)_2_ molecule detection model. (**B**) Real image of sample preparation and acquisition steps. (**C**) The hypothetical decision boundary learned by the proposed Radial Basis Function Kernel Support Vector Machine (RBFSVM) model.

In this study, we use lead (II) nitrate (Pb(NO_3_)_2_) as the target molecule. The Pb(NO_3_)_2_ is purchased from Sigma Aldrich (Yongin, Korea) and the molecule is prepared in deionized water. We use commercially available SERS substrates (SERSpace, Kwanglim Precision Co., Ltd., Daegu, Korea) for the measurements. The wavelength of the Raman spectrometer (NS200, Nanosystems Co., Ltd., Daejeon, Korea) is 785 nm, and the laser power and exposure time are fixed at 200 mW and 500 ms, respectively. To acquire the SERS spectra, we drop a 2.5-uL sample on the SERS substrate and dry it at room temperature (27 °C). To minimize signal degradation, each SERS measurement is taken with 10-s intervals (for each condition, total acquisition time is 1 h 40 min). Each measurement sample $S\in R^{1\times 2000}$ (the SERS spectrum) consists of 2000 wave-numbers (attributes). Figure 1 shows the experimental setup used for the measurement and classification of the (Pb(NO_3_)_2_) SERS dataset.

To ensure reproducibility of results, we performed two consecutive experiments, named as batch1 and batch2. In each batch, we had 500 negative $S_{N}\in R^{500\times 2000}$ and 1500 positive $S_{P}\in R^{1500\times 2000}$ samples. The concentration of ≥0.01 μM is used as the threshold for positive (detection) which is per the World Health Organization (WHO) heavy metals detection guideline [1]. In particular, we acquired 4 concentrations data. For both batches, we measured 0.01 μM, 0.1 μM, 10 μM, and 1000 μM. Although the concentrations are the same in each batch, some minor variations due to manual handling cannot be ruled out. For each batch and each concentration, the SERS measurements are acquired using a separate substrate. It ensures that the measurements have both the device and handling variability. This is important because the task of molecule concentration classification is trivial in single experiment (batch) (see, Section 3.4). Complete description of the sample distribution is furnished in Table 1.

### 2.2. Preprocessing

In machine learning-based model designing, data preprocessing is one of the crucial steps. In the proposed study, a Baseline Correction (BC) technique is used. Baseline correction refers to the removal of low-frequency components considered background noise [28]. In particular, we invoke Iterative Restrictive Least Square (IRLS) based baseline correction method proposed by [29]. We use the *baseline.irls* function in the *baseline* R package [30]. For comparison of the preprocessing method, we used RAW and normalized datasets. The normalization is the removal of sources of systematic variation between sample profiles to ensure that the spectra are comparable across related sample sets [31]. In particular, we consider Power Spectrum Density Normalization (PSN). The PSN for *j*-th wavenumber of *i*-th sample $S_{i,j}$ is defined as:

$$S{\mathrm{psn}}_{i,j}=\frac{S_{i,j}}{\Sigma S_{i,j}},$$

where Spsn is the power spectrum normalized signal and $\Sigma S_{i}$ is the sum of all intensity values for a sample $S_{i,j}$. We normalized the preprocessed dataset by the absolute maximum of each spectrum. Therefore, a preprocessed sample spectrum $S_{i}^{{}^{\prime }}$ is defined as:

$$S_{i,j}^{{}^{\prime }}=\frac{S_{i,j}}{\max(|S_{i}|)}.$$

### 2.3. Model Configuration, Training/Validation, and Test Protocol

To design a machine learning-based heavy-metal ion detector, we utilized the Radial-Basis-Function (RBF) kernel with the Support Vector Machine (SVM) classifier. The proposed Radial-Basis-Function Kernel support vector machine (RBFSVM) [32]) is trained using 80% data from a single batch. For data splitting, we used the stratified splitting method using the train test split function in the *scikit-learn* [33]) package. After model training, the remaining 20% is used for validation. Later, the trained model is used for the performance evaluation on the independent dataset (obtained from a different batch). We used $\ell_{2}$ penalty of C=1 and consider the kernel coefficient $\gamma =1/(2000\times \sigma_{s_{i}}^{2})$. Here, $\sigma_{s_{i}}^{2}$ stands for variance of the spectrum. The model is implemented using the *scikit-learn* package with default settings on Python 3.

### 2.4. Performance Evaluation

For performance evaluation of the proposed model, we used the Balanced Accuracy (BACC) as our primary performance metric supplemented with the other class-specific metrics such as sensitivity, specificity, F1 score, Matthews Correlation Coefficient (MCC), and Youden’s index. It is worthwhile mentioning that simple accuracy is not a suitable metric to quantify the performance due to the imbalanced nature of the dataset.

## 3. Results and Discussion

### 3.1. Performance Evaluation of the Proposed Model

For the performance evaluation, the proposed model is trained and tested for cross-batch datasets. We performed 10 independent trials and reported mean and standard deviations for each performance metric. Table 2 shows the individual and average results of different batches. The results are shown for the proposed model that is trained using RAW, PSN, and BC preprocessing techniques. From the results in Table 2, it is observed that the proposed method (BC + RBFSVM) has consistent performance for both batches and it outperforms the RAW and PSN preprocessing techniques. Overall, the proposed method (BC + RBFSVM) has achieved a 0.769, 0.805, 0.623, 0.846, and 0.692 average accuracy, F1-Score, MCC, BACC, and Youden’s Index, respectively, which in turn are 0.158, 0.152, 0.292, 0.173, 0.347, and 0.019, −0.052, 0.623, 0.346, 0.692 units higher than the RAW- and PSN-based implementations, respectively. It is worth noting that the model trained under PSN conditions only predicts positive classes regardless of the input data sets from both batches (100% sensitivity, 0% specificity for all cases). Therefore, the use of PSN + RBFSVM in the Pb(NO_3_)_2_ molecule detection task is not appropriate. However, the performance of PSN + RBFSVM in the same batch showed an average of 0.946 BACC.

### 3.2. Data Exploratory Analysis Using PCA-Aligned Cross-Batch Density-Preserving Data Visualization

To identify the source of improved performance achieved by the proposed baseline correction method, we analyze the RAW, PSN, and BC filtered data using the density-preserving data visualization technique called *D-tSNE* [34]. Unlike conventional t-SNE [35], the density preserving t-SNE, (*D-tSNE*), not only recovers Nearest-Neighborhood (NN) graph but also the spreading of individual data point using Kernel-Density Estimation (KDE) [34]. The *D-tSNE* is learned by minimizing the distance between original to embedding distributions using *Kullback-–Leibler* (KL) divergence while maximizing the correlation between original density radius and embedding density radius. In particular, we calculate the eigenvectors (principal components) first using the training dataset of one batch. The estimated principal components from one batch are used to project data from both batches in the common space. To visualize the preprocessed low-dimensional embedding of the Pb(NO_3_)_2_, the dimensions of the SERS measurement vectors $S\in R^{4000\times 2000}$ are reduced later on by mapping the PCA-aligned dataset to two-dimensional *D-tSNE* embedding $\Psi \in R^{4000\times 2}$.

**Figure 2:**
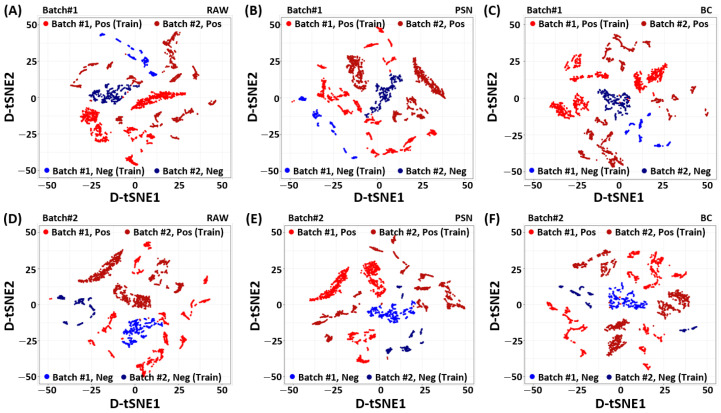
Visualization of the Density preserving t-SNE (*D-tSNE*) embedding of the Pb(NO_3_)_2_ SERS spectrum for (**A**,**D**) RAW, (**B**,**E**) Power Spectrum Density Normalization (PSN), and (**C**,**F**) Baseline Correction (BC) methods. The PCA embedding is learned with the 80% training dataset of one batch. The dataset of the other batch is projected using the learned PCA embedding. The *D-tSNE* is used as the dimension reduction technique to reduce the dimension of the projected dataset while keeping spreading of the data points. Top: Batch1. Bottom: batch2. Left: RAW. Middle: PSN. Right: BC.

Figure 2 shows the *D-tSNE* embedding of the SERS spectrum for each batch and class (positive/negative) of the Pb(NO_3_)_2_ according to the preprocessing methods. As shown in Figure 2 A and D, positive and negative samples of batch1 and batch2 respectively, are clustered in the raw data in a way that they can not be linearly separated in their respective classes. Therefore, we can not use a single classifier to separate positive and negative samples of both batches in the given raw data alone. It indicates that there exist some domain generalization (reputability/data variability) problems that can seriously affect the performance of the classifier on unseen data/batch.

To handle the aforementioned batch-effect, we investigate two different preprocessing techniques explained in Section 2. Figure 2B,E, show the PCA embedding of batch1 and batch2 using PSN. Although the PSN showed better alignment between two batches, however, it emphasizes the batch-effect. In contrast, the proposed BC shows desired batch-effect removal in Figure 2C,F, improves the class separability. It is noteworthy to point out that the PCA-aligned *D-tSNE* embedding [34] is an unsupervised data analysis method. It may not represent the actual class separability space (which is explored in the RBFSVM). However, it indicates the effect of preprocessing techniques and their importance for designing a reliable prediction model that can work for varying measurement conditions.

### 3.3. Comparative Analysis

For comparative analysis, we implemented six widely-used machine learning methods including Logistic Regression (LR) with ridge constraint (with $\ell_{2}$ penalty of C=1), Gaussian Naive Bayes (NB) [36]) with prior of 0.5 and 0.5, Decision Tree (DT) [37] with ‘Gini’ as a measure of impurity, Random Forest (RF) with 100 estimators, support vector machine with a linear kernel (LinSVM) [38,39], and Multi-Layer Perceptron (MLP) [40]. We compare the balanced accuracy of the proposed model with the aforementioned machine learning models. Table 3 shows the individual and average results of different batches. All models are implemented using the *scikit-learn* package [33] on Python 3, and are trained and tested for cross-batch datasets. The experiments are repeated for 10 independent trials and average results are reported with their standard deviation.

As shown in Table 3, the NB shows better performance compared to the proposed model for batch1 training and batch2 testing case. In contrast, the proposed model shows the best performance among all other models (BACC 0.934) for the batch2 training and batch1 testing case. Since the proposed model can learn nonlinear classification boundaries more efficiently than other models, it renders the best performance among all other models. In a nutshell, the proposed method shows a relatively consistent performance for both batches compared to all other models and achieves 0.846 BACC that is 0.188, 0.212, 0.057, 0.192, 0.152, and 0.021 units higher than the LR-, LinSVM-, NB-, DT-, RF-, and MLP-based implementations, respectively.

### 3.4. Discussion

The detection of the heavy metal ion (such as lead and chrome) by SERS measurements has been extensively studied [1]. However, the acquired SERS signal is difficult to analyze due to the inherent variability of each SERS device fabrication method and the nonlinearity of the signal. Towards this end, many studies have focused on fabricating reproducible devices to reduce measurement variabilities; see, for instance, Chan et al. [41], Wu et al. [13], and Cong et al. [42]. Unfortunately, little effort has been devoted to developing methods based on signal processing and machine learning.

Several applications of machine learning have been reported in the fields of SERS signal acquisition and data analysis [27,43,44]. However, the performance of the machine learning models according to the SERS preprocessing methods and the reproducibility according to the batch-effect has been rarely discussed. Towards this end, different normalization methods such as the PSN and BC were considered in this study to establish a benchmark for the performance evaluation of various machine learning models. In addition, two independent experimental batches were constructed to conduct training and independent evaluation to examine the reproducibility of the trained models. The combination of optimal model and preprocessing techniques for the Pb(NO_3_)_2_ molecule detection were derived by examining the variations in the model performance between batches through the independent test set evaluation. For each batch and each concentration, the SERS measurements were acquired using a separate substrate to ensure both device and handling variability of the measurements. It is important to note that the task of molecule concentration classification in a single experiment (batch) is trivial (See Table 4). Therefore to ensure reproducibility on unseen data, we design a cross-batch evaluation protocol.

In particular, we qualitatively evaluate the identification difficulty of the measured SERS data through cross-batch PCA-aligned density preserving t-SNE (*D-tSNE* [34]) embedding using different pre-processing techniques in Section 3.2. We also implemented 6 conventional machine learning models, specifically, Naive Bayes (NB), Decision Trees (DT), ridge Logistic Regression (LR), Random Forest (RF), the SVM with the linear kernel (LinSVM), and the Multi-Layer Perceptron (MLP). We compared the performance of these models with the proposed model. We evaluated the performance of these models on the BC dataset since it provides the best performance for the proposed model. As shown in Table 3, all models have performed poorly on independent datasets except the proposed model. It is observed that the MLP shows a relatively better performance than all other models. It may indicate that while the proposed model and MLP can handle some domain adaptation problems, all other models did not. The machine learning model implemented in this study can be considered relatively simple. It is expected that if the model is simple and works well under certain conditions, it can be the best model for the problem. We tested several advanced models, such as the DNN model but did not find any advantage over conventional machine learning models. Here we focus on the characterization of datasets, benchmarking the performance of several machine learning models, and finding the best model for a given problem. Since a firm baseline of the problem with the given study is derived, a more advanced model incorporating batch variation as a learnable parameter would be interesting and considered in the future.

## 4. Conclusions

In this study, an optimal preprocessing technique, model training, and evaluation protocol for the SERS-based Pb(NO_3_)_2_ molecule detection were proposed. Moreover, a benchmark dataset and python code are provided to lay the foundation for advanced model construction and evaluation. The proposed model showed excellent performance on the Pb(NO_3_)_2_ molecule detection task compared to other machine learning models. We believe that these results could be used as a benchmark for further development of the SERS measurements-based advanced heavy metal ion-molecule detection models, such as end-to-end deep learning models.

## Figures and Tables

**Table 1:** Sample statistics of Pb(NO_3_)_2_.

	Negative	Positive		
Concentration (uM)	0.01	0.1	10	1000
Batch1	500	500	500	500
Batch2	500	500	500	500

**Table 2:** Performance summary of the proposed model.

Dataset	Train	Test	Accuracy	Sensitivity	Specificity	F1	MCC	BACC	Youden’s Index
RAW	Batch1	Batch2	0.501 ± 0.000	0.335 ± 0.000	1.000 ± 0.000	0.501 ± 0.000	0.334 ± 0.000	0.667 ± 0.000	0.335 ± 0.000
	Batch2	Batch1	0.721 ± 0.019	0.765 ± 0.011	0.591 ± 0.084	0.805 ± 0.011	0.328 ± 0.069	0.678 ± 0.040	0.356 ± 0.080
	Average		0.611 ± 0.114	0.550 ± 0.221	0.796 ± 0.218	0.653 ± 0.156	0.331 ± 0.048	0.673 ± 0.028	0.345 ± 0.056
PSN	Batch1	Batch2	0.750 ± 0.000	1.000 ± 0.000	0.000 ± 0.000	0.857 ± 0.000	0.000 ± 0.000	0.500 ± 0.000	0.000 ± 0.000
	Batch2	Batch1	0.750 ± 0.000	1.000 ± 0.000	0.000 ± 0.000	0.857 ± 0.000	0.000 ± 0.000	0.500 ± 0.000	0.000 ± 0.000
	Average		0.750 ± 0.000	1.000 ± 0.000	0.000 ± 0.000	0.857 ± 0.000	0.000 ± 0.000	0.500 ± 0.000	0.000 ± 0.000
Proposed (BC+RBFSVM)	Batch1	Batch2	0.637 ± 0.005	0.517 ± 0.007	0.999 ± 0.001	0.681 ± 0.006	0.459 ± 0.004	0.758 ± 0.003	0.516 ± 0.006
	Batch2	Batch1	0.901 ± 0.005	0.868 ± 0.006	1.000 ± 0.000	0.929 ± 0.004	0.788 ± 0.008	0.934 ± 0.003	0.868 ± 0.006
	Average		0.769 ± 0.135	0.692 ± 0.180	1.000 ± 0.001	0.805 ± 0.127	0.623 ± 0.169	0.846 ± 0.09	0.692 ± 0.180

**Table 3:** Performance comparison between the proposed model and six ML models. The proposed model showed the best independent test balanced accuracy (BACC) results for datasets corresponding to both batches.

Model	Train/Test		Average
	Batch1/Batch2	Batch2/Batch1	
LR	0.735 ± 0.005	0.582 ± 0.008	0.658 ± 0.079
LinSVM	0.661 ± 0.042	0.606 ± 0.011	0.634 ± 0.042
NB	0.882 ± 0.002	0.695 ± 0.006	0.789 ± 0.096
DT	0.574 ± 0.031	0.733 ± 0.072	0.654 ± 0.098
RF	0.603 ± 0.006	0.784 ± 0.047	0.694 ± 0.098
MLP	0.754 ± 0.006	0.896 ± 0.030	0.825 ± 0.076
RBFSVM	0.758 ± 0.003	0.934 ± 0.003	0.846 ± 0.090

**Table 4:** Ten-folds cross validation performance using same batch datasets.

Dset	Train	Test	Accuracy	Sensitivity	Specificity	F1	MCC	BACC	Youden’s Index
RAW	Batch1	Batch1	0.999 ± 0.002	0.999 ± 0.003	1.000 ± 0.000	0.999 ± 0.001	0.997 ± 0.006	0.999 ± 0.001	0.999 ± 0.003
	Batch2	Batch2	0.922 ± 0.015	0.906 ± 0.020	0.972 ± 0.036	0.946 ± 0.011	0.820 ± 0.034	0.939 ± 0.018	0.878 ± 0.036
	Average		0.961 ± 0.041	0.952 ± 0.050	0.986 ± 0.028	0.973 ± 0.028	0.909 ± 0.094	0.969 ± 0.033	0.938 ± 0.067
PSN	Batch1	Batch1	1.000 ± 0.002	0.999 ± 0.002	1.000 ± 0.000	1.000 ± 0.001	0.999 ± 0.004	1.000 ± 0.001	0.999 ± 0.002
	Batch2	Batch2	0.900 ± 0.037	0.906 ± 0.033	0.880 ± 0.064	0.931 ± 0.026	0.751 ± 0.089	0.893 ± 0.044	0.786 ± 0.089
	Average		0.950 ± 0.057	0.953 ± 0.053	0.940 ± 0.076	0.965 ± 0.040	0.875 ± 0.141	0.946 ± 0.063	0.893 ± 0.125
BC	Batch1	Batch1	1.000 ± 0.000	1.000 ± 0.000	1.000 ± 0.000	1.000 ± 0.000	1.000 ± 0.000	1.000 ± 0.000	1.000 ± 0.000
	Batch2	Batch2	0.973 ± 0.012	0.986 ± 0.009	0.934 ± 0.038	0.982 ± 0.008	0.928 ± 0.032	0.960 ± 0.020	0.920 ± 0.039
	Average		0.986 ± 0.016	0.993 ± 0.009	0.967 ± 0.043	0.991 ± 0.011	0.964 ± 0.043	0.980 ± 0.025	0.960 ± 0.049

## Data Availability

The code and dataset utilized in this work are available at the author’s GitHub (https://github.com/psychemistz/sersml (accessed on 20 November 2021).
